# Causative Agents of Ventilator-Associated Pneumonia and Resistance to Antibiotics in COVID-19 Patients: A Systematic Review

**DOI:** 10.3390/biomedicines10061226

**Published:** 2022-05-24

**Authors:** Larry Velásquez-Garcia, Ana Mejia-Sanjuanelo, Diego Viasus, Jordi Carratalà

**Affiliations:** 1 Department of Medicine, Division of Health Sciences, Universidad del Norte and Hospital Universidad del Norte, Barranquilla 081001, Colombia; larryv@uninorte.edu.co (L.V.-G.); anammejia629@gmail.com (A.M.-S.); dviasus@uninorte.edu.co (D.V.); 2 Department of Infectious Diseases, Bellvitge University Hospital—Bellvitge Biomedical Research Institute (IDIBELL), University of Barcelona, 08907 Barcelona, Spain; 3 Centro de Investigación Biomédica en Red de Enfermedades Infecciosas (CIBERINFEC), Instituto de Salud Carlos III, 28029 Madrid, Spain

**Keywords:** COVID-19, SARS-CoV-2, ventilator-associated pneumonia, causative pathogens, microbiology, antibiotic resistance

## Abstract

Patients with coronavirus disease 2019 (COVID-19) have an increased risk of ventilator-associated pneumonia (VAP). This systematic review updates information on the causative agents of VAP and resistance to antibiotics in COVID-19 patients. We searched the Cochrane Central Register of Controlled Trials (CENTRAL), PubMed/MEDLINE, and LILACS databases from December 2019 to December 2021. Studies that described the frequency of causative pathogens associated with VAP and their antibiotic resistance patterns in critically ill COVID-19 adult patients were included. The Newcastle-Ottawa Quality Assessment Scale was used for critical appraisal. The data are presented according to the number or proportions reported in the studies. A total of 25 articles were included, involving 2766 VAP cases in COVID-19 patients (range 5–550 VAP cases). Most of the studies included were carried out in France (32%), Italy (20%), Spain (12%) and the United States (8%). Gram-negative bacteria were the most frequent causative pathogens of VAP (range of incidences in studies: *P. aeruginosa* 7.5–72.5%, *K. pneumoniae* 6.9–43.7%, *E. cloacae* 1.6–20% and *A. baumannii* 1.2–20%). *S. aureus* was the most frequent Gram-positive pathogen, with a range of incidence of 3.3–57.9%. The median incidence of *Aspergillus* spp. was 6.4%. Few studies have recorded susceptibility patterns among Gram-negative causative pathogens and have mainly reported extended-spectrum beta-lactamase (ESBL), AmpC, and carbapenem resistance. The median frequency of methicillin resistance among *S. aureus* isolates was 44.4%. Our study provides the first comprehensive description of the causative agents and antibiotic resistance in COVID-19 patients with VAP. Gram-negative bacteria were the most common pathogens causing VAP. Data on antibiotic resistance patterns in the published medical literature are limited, as well as information about VAP from low- and middle-income countries.

## 1. Introduction

The severe acute respiratory syndrome coronavirus-2 (SARS-CoV-2) pandemic has been associated with high mortality and serious socio-economic consequences [1]. One study found an aggregated coronavirus disease 2019 (COVID-19) infection fatality rate of 0.68% (95% confidence interval 0.53–0.82%) [2]. Although in most patients the illness is mild, those with comorbidities and older patients present a higher risk of poor prognosis [1,3]. Importantly, among those cases requiring intensive care unit (ICU) admission and mechanical ventilation, a meta-analysis documented a pooled mortality of 28.3% and 43%, respectively [4]. However, analyses have found major regional differences in COVID-19 outcomes.

Several studies have reported an increased frequency of healthcare-associated infections in COVID-19 patients. Ventilator-associated pneumonia (VAP), bloodstream infection (BSI), and catheter-related BSI are the most frequently recorded healthcare-associated infections [5,6]. A study evaluating the impact of COVID-19 on BSIs before and during the pandemic in 69 US hospitals [5] found that COVID-19 infected patients were 3.5 times more likely to develop hospital BSI than those without COVID-19 infection. Moreover, after adjusting for confounders in a multivariate analysis, DeVoe et al. [6] found that rates of probable VAP were nearly 10 times higher in COVID-19 patients than in inpatients without positive SARS-CoV-2 or influenza.

Many of the causative pathogens of VAP are part of the endogenous microflora, while those associated with cross-transmission or reservoirs are less common. Significantly, multidrug-resistant (MDR) organisms have been found in up to one third of all healthcare-associated infections. The clinical importance of determining the frequency of MDR organisms is based on their virulence and increased resistance to antibiotics, leading to a higher risk of inappropriate empirical therapy and negatively affecting patient outcomes [7,8]. Comprehensive data on the global epidemiology of MDR pathogens in patients with VAP are lacking. One study found a broad difference in the prevalence of MDR *A. baumannii* causing HAP and VAP between countries [9]. Central and Latin America had the highest prevalence, whereas Eastern Asia had the lowest. Bacteria and fungi have been documented as causative pathogens of VAP in COVID-19 patients. However, information about antimicrobial susceptibility in COVID-19 patients with VAP is scarce. Importantly, studies have found a high antimicrobial consumption among COVID-19 patients [10,11]. This may have an impact on patient safety, increasing the risk of resistant infections [11].

Thus, this systematic review updates information on the causative agents of VAP in COVID-19 patients and their patterns of antibiotic resistance.

## 2. Materials and Methods

This study was performed in accordance with the Preferred Reporting Items for Systematic reviews and Meta-Analyses (PRISMA) guidelines [12]. We searched the Cochrane Central Register of Controlled Trials (CENTRAL), PubMed/MEDLINE, and LILACS (Literatura Latino-Americana e do Caribe de Informação em Ciências da Saúde) databases from December 2019 to December 2021 for studies that described the frequency of causative pathogens associated with VAP and their antibiotic resistance patterns in critically ill COVID-19 patients. Relevant publications from reference lists of reviews were also evaluated. We included observational studies (cohort studies and case-control studies) and randomized controlled trials available in English and Spanish, excluding studies of animals and pediatric populations, abstracts, and case reports. We used the keywords “ventilator-associated pneumonia OR nosocomial pneumonia” AND “COVID-19 OR SARS-CoV-2 OR coronavirus” in the search in the databases. This study is not registered in the International Registry of Systematic Reviews/Meta-Analyses due to an error during the submission. However, it has been adjusted to relevant ethical guidelines.

Two authors (LAV and AMM) independently screened the titles and/or abstracts to identify studies that potentially met the criteria outlined above. The full text of these potentially eligible studies was independently assessed for eligibility. Any disagreement was resolved by a third reviewer (DV). Using a standardized form created for the review, the following information was extracted from the included articles: author, year, journal, the country in which the study was conducted, original study design, sample size, sample size subgroups (critical ill and invasive mechanical ventilated patients), causative pathogens, resistance patterns, and outcome (mortality). The Newcastle-Ottawa Quality Assessment Scale was used for critical appraisal of the studies. All quality assessments were performed independently by two reviewers (LAV and AMM). Disagreements were resolved by a consensus-based discussion.

The data are presented according to the number or proportions reported in the studies of the incidence of the causative pathogens and the frequency of the resistance patterns. When possible, the median of these percentages was calculated together with the range or interquartile range of the studies that reported isolates of the causative pathogens. All results were analyzed using IBM SPSS 23.0 (SPSS Inc., Chicago, IL, USA).

## 3. Results

**Figure 1:**
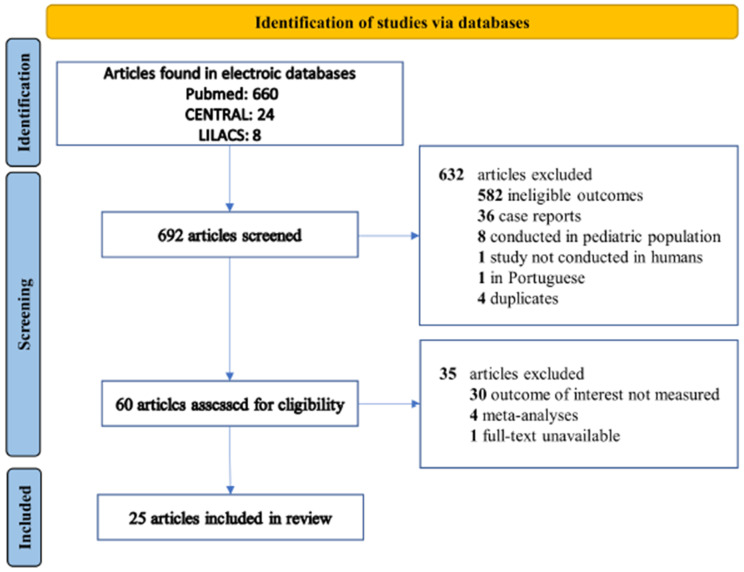
Study selection flow diagram.

Our initial search strategy yielded 692 articles of interest. After excluding 632 due to duplication or because they did not meet the inclusion criteria based on the title and abstract, we found 60 relevant articles that were considered for full-text review and eligibility. Finally, 25 articles that met the criteria were included in this systematic review [13,14,15,16,17,18,19,20,21,22,23,24,25,26,27,28,29,30,31,32,33,34,35,36,37]. The flow chart of the systematic literature search according to PRISMA guidelines is shown in Figure 1.

### 3.1. Study Characteristics and Quality

The 25 articles included had an observational design (prospective and retrospective cohort studies) (Table 1) and involved 2766 VAP cases in COVID-19 patients (range 5–550 cases). Most studies included patients with immunosuppression (active solid cancer, hematologic cancer, organ transplant, HIV, or immunosuppressive drugs). VAP etiology was identified by culture in 22 studies and by culture and molecular tests in three. Most of the studies included were carried out in France (32%), Italy (20%), Spain (12%) and the United States (8%). Definition of VAP was described in the methodology section of 22 (88%) of the studies. Ten studies reported VAP mortality. The range of mortality in COVID-19 patients with VAP was from 9.3% to 59.5% (median 43.2%). The risk of bias among the 25 studies was generally low according to The Newcastle-Ottawa Quality Assessment Scale (Table 2).

### 3.2. VAP Caused by Gram-Negative Pathogens

The identification of *Klebsiella* spp., *K. oxytoca*, *K. variicola*, or *K. pneumoniae* was reported in 18 (72%) of 25 studies. *K. pneumoniae* was reported in 12 (48%). The range of frequency of *K. pneumoniae* identification among these studies was 6.9–43.7% (median 13.7%). Two studies reported resistance patterns of *K. pneumoniae*. In one study, pan-drug resistant (PDR) *K. pneumoniae* was documented in all isolations [19]. Likewise, Moretti et al. [33] found that 71.4% of *K. pneumoniae* had extended-spectrum beta-lactamase (ESBL). Moreover, *Acinetobacter* spp. or *A. baumannii* were found in 15 (60%) of the included studies. *A. baumannii* was described in 11 (44%), and the range of incidence was 1.2–20% (median 4.3%). The resistance pattern of *A. baumannii* was reported in three studies [19,24,36]. All *A. baumannii* were multidrug-resistant (MDR), defined as resistant to ≥ one drug in at least three classes of antibiotics.

*Pseudomonas aeruginosa* was identified as the causative pathogen of VAP in 22 of the 25 studies included (median frequency of isolation 19%, range 7.5–72.5%). Most studies did not report the resistance patterns of this microorganism. Vacheron et al. [37] reported resistance to carbapenems in 42 (21.5%) of 195 *P. aeruginosa* isolated from VAP cases, and another study found that all isolated *P. aeruginosa* had an ESBL resistance pattern [19]. VAP caused by *Enterobacter cloacae* was reported in 11 (44%) of the studies. The range of incidence was 1.6–20% (median 6.9%). The resistance pattern was reported in one study. Luyt et al. [27] found that all these causative pathogens were inducible AmpC-cephalosporinase producers.

Other Gram-negative causative pathogens isolated in the studies included *Escherichia coli*, *Serratia marcescens*, *Proteus* spp., *Morganella morganii*, *Stenotrophomonas maltophilia*, *Citrobacter* spp., *Haemophilus influenzae*, *Rautella Ornithyolytica*, *Moraxella catarrhalis*, and *Burkholderia cepacia*.

### 3.3. VAP Caused by Gram-Positive Pathogens

VAP caused by *Enterococcus* spp. was reported in 9 (36%) of the studies. Among these studies, the range of the frequency of isolation of *Enterococcus* spp. was 1.3–16.1% (median 6.2%). *E. faecium* was identified in bronchoalveolar lavage culture in one study, in 5 (8.06%) out of 62 cases of VAP [16]. The resistance pattern was not reported. For its part, *Staphylococcus aureus* was identified in 21 (84%) of the 25 studies. The range of incidence of *S. aureus* as causative pathogen of VAP was 3.3–57.9% (median 15.8%). Resistance patterns were reported in 15 (78.1%) of 22 studies, and methicillin-resistant strains of *S. aureus* (MRSA) were frequent (median 44.4%).

Other Gram-positive causative pathogens isolated in studies included *Streptococcus* spp., *Stomatococcus* spp., *Streptococcus pneumoniae*, *Streptococcus agalactiae*, *Streptococcus viridans*, and *Corynebacterium accolens*.

### 3.4. Fungal VAP

The isolation of *Candida albicans*, *Candida glabrata*, *Candida krusei*, *Candida auris*, *Aspergillus fumigatus*, *Aspergillus niger*, *Aspergillus terreus*, *Aspergillus* spp. and *Mucor* spp. was documented in seven studies (28%). *Aspergillus* spp. was documented in the same number of articles. The median incidence of *Aspergillus* spp. among studies that reported fungi was 6.4% (range 2–40%).

## 4. Discussion

This systematic review describes the causative agents and antibiotic resistance patterns of 2766 VAP cases in COVID-19 patients. Gram-negative bacteria were the most frequent causative pathogens. *S. aureus* also was a frequent etiology of VAP in COVID-19 patients. However, information on resistance patterns was scarce. The few studies that reported susceptibility patterns found a high frequency of antibiotic resistance among causative pathogens: for example, ESBL, AmpC and carbapenem resistance among Gram-negative bacteria. Similarly, the frequency of methicillin-resistance among *S. aureus* was high.

VAP is one of the commonest healthcare-associated infections, resulting in prolonged hospitalization with increased morbidity and mortality among patients in the ICU. Certain patient characteristics, prolonged time of mechanical ventilation and hospital stay, altered state of consciousness, prior antibiotic therapy and gene polymorphisms have been related to the development of VAP [38]. Moreover, a meta-analysis found considerable variation in mortality rates in VAP (14% to 78%) in non-COVID-19 patients. VAP was associated with higher mortality (relative risk 1.27, 95% CI 1.15–1.39), although with considerable heterogeneity between studies [39]. Another study documented an attributable mortality of 13% in VAP. Higher mortality rates were associated with severity scores at admission and with surgery [40]. In the present systematic review, we found a median mortality rate of 43.2% (range 9.3–59.5%) in COVID-19 patients with VAP, a similar value to that reported in non-COVID-19 patients. In addition, our mortality coincides with that reported in a recent meta-analysis that evaluated mortality in patients with COVID-19 and VAP (pooled mortality was 42.7%, 95% C.I. 34–51.7%) [41].

In the present study, *P. aeruginosa*, *K. pneumoniae*, *E. cloacae* and *A. baumannii* were the main Gram-negative causative pathogens (nearly half of all VAP cases). However, *S. aureus* was also a frequent etiology of VAP (15.8% of all VAP cases). Notably, we did not find differences in the incidence of causative pathogens of VAP in COVID-19 patients between European countries and the US (data not shown). Our findings concur with studies of VAP in non-COVID-19 patients which found that despite considerable geographical variations these pathogens account for most of the VAP episodes [8]. The etiology in non-COVID-19 patients has been studied in depth, with *Enterobacteriacae* spp. appearing as the most common pathogen, followed by *Pseudomonas aeruginosa* and *Staphylococcus aureus*, with relatively minor changes in the distribution of the pathogens over the last decade [42]. Significantly, the World Health Organization (WHO) recently published a list which includes some of these pathogens that represent a global challenge, due to their increasing incidence, the difficulty of their treatment, and their impact on outcomes [43].

Information on antimicrobial resistance patterns in causative pathogens of VAP in COVID-19 patients is scarce. In addition, the few studies that have described resistance patterns have done so using different criteria. Among studies that described antibiotic resistance, high frequencies of ESBL, AmpC, and carbapenem resistance were found in Gram-negative bacteria, especially in *K. pneumoniae* and *P. aeruginosa*. Similarly, the frequency of methicillin-resistance among *S. aureus* was high. Therefore, VAP in COVID-19 patients complicates treatment processes due to both the viral infection and antibiotic resistance. Recent studies have reported an increase in the frequency of healthcare-associated infections and antibiotic resistance during the COVID-19 pandemic. In Mexico, López-Jácome et al. [44] found that antimicrobial resistance rose during the COVID-19 pandemic; they reported increases in oxacillin resistance for *S. aureus* and carbapenem resistance for *K. pneumoniae*, and increased resistance rates for *A. baumannii* and *P. aeruginosa*. Another study found a significant difference in the resistance for ceftazidime and levofloxacin in *P. aeruginosa* strains from endotracheal aspirate cultures of critically ill COVID-19 patients compared to the pre-pandemic period [45]. Similarly, antibiotic resistance rates in *A. baumannii* strains also rose following the pandemic.

Recent studies have identified COVID-19 as a risk factor associated with invasive pulmonary aspergillosis. Among COVID-19 patients receiving invasive mechanical ventilation, the incidence of invasive pulmonary aspergillosis has been reported to be as high as 28% [46,47,48], and it was also associated with prolonged ICU stay and higher mortality [46]. In the present systematic review, the rate of isolation of *Aspergillus* spp. across the studies was 6.4%. However, most studies did not specify whether investigators used systematic screening for fungi, and the failure to do so may have led to an underestimation of the overall incidence of invasive pulmonary aspergillosis. In addition, these studies included immunosuppressed patients: in this connection, Fekkar et al. [47] found a low risk of invasive pulmonary fungal infection, especially aspergillosis, in patients with no underlying immunosuppression and severe COVID-19.

Our study has several limitations. First, causative agents of VAP in patients with COVID-19 and resistance patterns may vary between health institutions, cities, and countries. Therefore, the local causative pathogens and the resistance pattern of each health institution must be borne in mind in the selection of initial empiric antibiotics, and physicians should assess risk factors for multidrug-resistant pathogens. Timely identification of these pathogens can improve outcomes. Second, the studies in this systematic review included heterogeneous populations due to different risk factors for causative pathogens, such as immunosuppressed patients. The available information does not allow an analysis of the differences in the incidence of the causative pathogens and resistance phenotypes between these groups. Third, as most studies were carried out in European countries or the United States, the data may not represent the epidemiological situation in countries with medium or low resources. In this regard, we only included studies in the English or Spanish languages, which could lead to selection bias. Fourth, the studies only rarely describe the methodology used to identify and report isolates and their resistance patterns. Finally, most studies do not provide information regarding the time of occurrence of VAP, and the causative pathogens may vary depending on whether the occurrence is early or late after intubation.

## 5. Conclusions

Our study provides the first comprehensive description of the causative pathogens and VAP resistance patterns in COVID-19 patients. Gram-negative bacteria, such as *P. aeruginosa*, *K. pneumoniae*, *E. cloacae*, and *A. baumannii*, were the most common pathogens causing VAP. However, little information was available on resistance patterns. ESBL, AmpC and carbapenem resistance among Gram-negative bacteria was frequent. Similarly, the frequency of methicillin resistance among *S. aureus* was high. Interestingly, VAP etiology does not seem to differ between COVID-19 and non-COVID-19 patients. Further studies are needed to assess the incidence of causative pathogens and resistance patterns between groups, for instance between immunosuppressed and non-immunosuppressed patients. In addition, more information from low- and middle-income countries is necessary.

## Figures and Tables

**Table 1:** Characteristics of studies included evaluating etiology of VAP in COVID-19 patients.

First Author	Year	No. of Patients Requiring MV	No. of Patients with VAP	Immunosuppression	VAP Mortality	Main Causative Pathogens
Beaucote V, et al. [13]	2021	161	119	Corticosteroids 61%, immunosuppression (active solid cancer, hematologic cancer, organ transplant, HIV, or immunosuppressive drugs) 17%.	No information	*P. aeruginosa* (8%) *K. aerogenes* (4%) *S. aureus* (3%)
Gragueb–Chatti I, et al. [14]	2021	151	127	Immunosuppression 7%, history of neoplasm 12%, hydrocortisone for septic shock 18%, dexamethasone 55.6%.	No information	*S. aureus* (22%) *P. aeruginosa* (17%) *K. aerogenes* (9%) *K. pneumoniae* (8%)
De Pascale G, et al. [15]	2021	No information	40	Immunosuppression 6.7%, neoplasm 5.8%.	No information	*S. aureus* (100%)
De Santis V, et al. [16]	2021	No information	62	Neoplasm 6%, immunosuppression 3.6%, tocilizumab 28.6%.	No information	*P. aeruginosa* (22%) *S. aureus* (16%) *E. coli* (13%) *K. pneumoniae* (12%)
Karolyi M, et al. [17]	2021	60	48	Not specified.	36.7%	*S. aureus* (22%) *K. pneumoniae* (20%) *H. influenzae* (10%)
Luque-Paz D, et al. [18]	2021	178	66	Not specified.	28.3%	*Enterobacteriaceae* (65%) *S. aureus* (16%) *P. aeruginosa* (15%)
Meawed T, et al. [19]	2021	No information	331	Steroids 100%, tocilizumab 90%.	No information	*K. pneumoniae* (24.4%) *C. albicans* (17%) *A. baumannii* (16%) *P. aeruginosa* (12%)
Pickens C, et al. [20]	2021	179	72	Solid organ transplant 6.3%, bone marrow transplantation/malignancy 1.7%, other cancer 8.4%, anti-IL-6r 25.3%, corticosteroids 32%.	No information	*S. aureus* (18%) *S. viridans* (13%)
Risa E, et al. [21]	2021	126	69	Not specified.	No information	*S. aureus* (57%) *K. aerogenes* (15%)
Rouyer M, et al. [22]	2021	79	42	Corticosteroids 35%, neoplasm 5%, immunosuppressive treatment 5%.	81%	*Enterobacteriaceae* (54%) *P. aeruginosa* (19%)
Signorini L, et al. [23]	2021	92	75	Steroids 98%.	No information	*P. aeruginosa* (34%) *S. maltophila* (18%) *Enterococcus* spp. (14%)
Bardi T, et al. [24]	2021	134	21	Steroids 90%, tocilizumab 68%.	No information	*P. aeruginosa* (38%) *S. aureus* (23%)
Blonz G, et al. [25]	2021	194	92	Cancer 5.9%, hematologic malignancies 2.7%, HIV 2.1%, immunosuppressive therapy 3.7%, long-term corticosteroid therapy 1.6%.	30%	*P. aeruginosa* (33%) *S. aureus* (30%) *E. coli* (28%) *K. pneumoniae* (17%)
Suarez-de-la-Rica A, et al. [26]	2021	107	35	Solid tumor 10.3%, HIV 0.9%, corticosteroids 76.6%, tocilizumab 46.7%.	No information	*P. aeruginosa* (31%) *Klebsiella* spp. (25%)
Luyt C, et al. [27]	2020	54	43	Immunocompromised 2%.	9%	*P. aeruginosa* (37%) *K. aerogenes* (25%) *E. cloacae* (7%) *S*. *aureus* (7%)
Garcia-Vidal C, et al. [28]	2021	44	11	Cancer 8.4%, tocilizumab 21.8%, methylprednisolone 26%, dexamethasone 2.5%.	No information	*S. aureus* (36%) *P. aeruginosa* (27%)
Giacobbe DR, et al. [29]	2021	586	171	Solid cancer 6%, hematological malignancy 2%, steroids 63%, anti-IL-6 64%, anti-IL-1 3%.	45%	*P. aeruginosa* (15%) *S. aureus* (10.5%) *K. pneumoniae* (8.7%)
Grasselli G, et al. [30]	2021	688	389	Immunological comorbidity 12%, tocilizumab 24%.	No information	*S. aureus* (28%) *P. aeruginosa* (21%) *Klebsiella species* (11%)
Maes M, et al. [31]	2021	82	39	Immunocompromised 15%, corticosteroid use 13%.	No information	*P. aeruginosa* (14%) *E. coli* (14%) *K. pneumoniae* (12%)
Martinez-Guerra B, et al. [32]	2021	No information	69	Immunosuppression 5.7%, HIV 1.3%, steroid 9.2%.	No information	*P. aeruginosa* (14%) *Klebsiella* spp. (13%) *E. coli* (13%)
Moretti M, et al. [33]	2021	39	27	Immunosuppressant use 7.8%, neoplasm 7.8%.	40%	*K. pneumoniae* (25%) *P. aeruginosa* (18%)
Razazi K, et al. [34]	2020	90	58	Solid cancer 5%, blood cancer 21%, organ transplant 11%, HIV 5%, sickle cell disease 2%, others 6%, corticosteroids 37%.	56%	*Enterobacter* spp. (39%) *E. coli* (17%)
Rouze A, et al. [35]	2021	568	205	Immunosuppression 9.3%, corticosteroids 37%.	No information	*P. aeruginosa* (31%) *Enterobacter* spp. (26%) *S. aureus* (17%)
Sogaard KK, et al. [36]	2021	34	5	Not specified.	No information	*A. fumigatus* (40%)
Vacheron CH, et al. [37]	2021	No information	550	Not specified.	No information	*P. aeruginosa* (22%) *Enterobacter* spp. (14%) *Klebsiella* spp. (10%) *S. aureus* (10%)

Abbreviations: HIV, human immunodeficiency virus; MV, mechanical ventilation; VAP, ventilator-associated pneumonia.

**Table 2:** Risk of bias analysis of studies included evaluating etiology of VAP in COVID-19 patients.

	Selection	Comparability *	Outcome	Overall
Beaucote V, et al. [13]	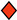 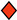 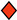	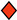 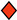	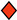 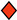 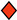	8
Gragueb–Chatti I, et al. [14]	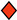 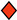 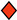	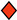 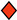	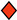 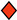 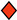	8
De Pascale G, et al. [15]	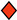 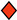 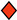	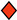 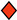	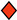 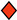 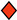	8
De Santis V, et al. [16]	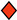 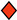 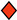	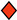 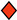	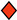 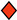 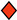	8
Karolyi M, et al. [17]	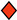 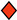 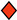	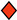	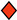 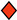 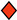	7
Luque-Paz D, et al. [18]	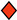 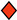 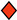	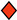	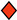 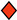 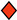	7
Meawed T, et al. [19]	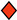 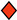 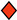	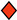 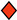	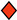 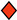 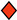	8
Pickens C, et al. [20]	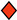 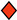 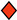	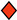 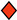	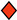 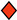 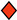	8
Risa E, et al. [21]	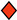 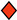 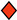	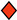	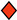 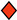 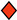	7
Rouyer M, et al. [22]	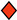 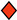 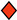 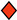	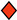 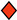	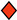 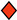 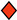	9
Signorini L, et al. [23]	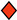 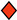 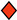	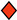	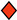 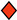 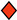	7
Bardi T, et al. [24]	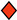 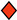 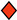	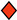 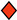	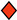 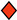 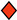	8
Blonz G, et al. [25]	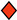 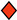 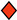	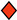 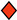	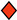 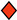 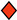	8
Suarez-de-la-Rica A, et al. [26]	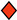 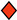 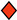	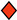	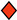 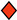 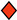	7
Luyt C, et al. [27]	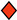 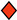	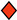 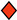	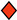 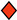 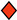	7
Garcia-Vidal C, et al. [28]	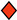 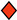 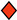	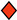 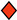	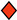 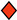 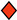	8
Giacobbe DR, et al. [29]	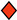 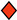 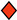	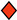 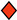	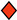 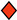 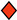	8
Grasselli G, et al. [30]	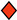 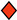 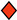	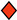 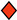	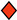 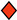 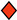	8
Maes M, et al. [31]	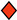 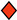 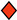 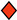	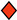 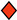	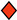 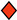 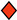	9
Martinez-Guerra B, et al. [32]	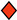 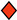 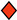	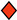 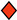	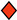 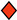 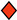	8
Moretti M, et al. [33]	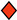 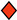 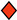	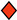 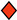	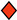 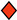 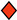	8
Razazi K, et al. [34]	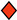 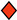 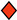 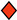	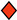 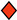	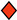 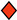 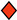	9
Rouze A, et al. [35]	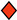 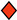 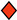 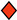	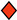 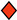	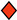 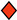 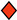	9
Sogaard KK, et al. [36]	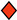 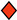 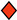	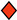	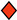 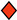 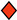	7
Vacheron CH, et al. [37]	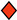 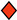 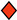 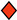	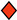	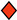 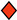 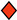	8

The Newcastle-Ottawa scale contains 8 items within 3 domains: the total maximum score is 9. A study with a score from 7–9 is rated as good quality, 4–6, high risk, and 0–3 very high risk of bias. * (a) Most important factor of adjustment (described immunosuppression); (b) Any additional factors (described days of invasive mechanical ventilation, duration of the COVID-19, or decontamination measures to reduce colonization). Abbreviations: VAP, ventilator-associated pneumonia.

## Data Availability

The data presented in this study are available on request from the corresponding author.
